# The UK experience of promoting dementia recognition and management in primary care

**DOI:** 10.1007/s00391-016-1175-1

**Published:** 2017-01-17

**Authors:** Steve Iliffe, Jane Wilcock

**Affiliations:** University College London, Gower Street, WC1E 6BT London, UK

**Keywords:** Dementia, Early diagnosis, Education, Financial incentives, Memory clinics, Demenz, Früherkennung, Bildung, Leistungszuzahlungen, Gedächtniskliniken

## Abstract

**Background:**

The early and timely recognition of dementia syndrome is a policy imperative in many countries. In the UK the achievement of earlier and timelier recognition has been pursued through educational interventions, incentivisation of general practitioners and the promotion of a network of memory clinics.

**Objective:**

The effectiveness of education, incentivisation and memory clinic activity are unknown. This article analyses data from different sources to evaluate the impact of these interventions on the incidence and prevalence of dementia, and the diagnostic performance of memory clinics.

**Material and methods:**

Three data sources were used: 1) aggregated, anonymised data from a network of general practices using the same electronic medical record software, The Health Information Network (THIN), 2) UK Health & Social Care Information Centre data reports and 3) Responses to Freedom of Information Act requests.

**Results:**

Educational interventions did not appear to change the recorded incidence of dementia syndrome. There was no apparent effect of education, incentives or memory clinic activity on the reported incidence of dementia syndrome between 1997 and 2011 but there were signs of change in the documentation of consultations with people with dementia. There was no clear impact of incentivisation and memory clinic activity in prevalence data. Memory clinics are seeing more patients but fewer are being diagnosed with dementia.

**Conclusion:**

It is not clear why there has been no upturn in documented incidence or prevalence of dementia syndrome despite substantial efforts and this requires further investigation to guide policy changes. The performance of memory clinics also needs further study.

## Zusammenfassung

**Hintergrund:**

Die früh- bzw. rechtzeitige Erkennung des Demenzsyndroms ist eine zwingend notwendige Strategie für viele Länder. In Großbritannien wird die Früherkennung durch pädagogische Intervention, Leistungszuzahlung für Allgemeinmediziner und die Förderung von Netzwerken von Gedächtniskliniken betrieben.

**Ziel der Arbeit:**

Die Auswirkungen der pädagogischen Interventionen, Leistungszuzahlungen und der Aktivität der Gedächtniskliniken sind nicht bekannt. Dieser Beitrag analysiert Daten aus verschiedenen Quellen, um die Auswirkungen dieser Maßnahmen auf die Inzidenz und Prävalenz von Demenz und die Diagnoseleistung von Gedächtniskliniken zu bewerten.

**Materialien und Methoden:**

Drei Datenquellen wurden verwendet: 1) aggregierte, anonymisierte Daten aus einem Netzwerk von allgemeinen Praxen, welche die gleiche elektronische Patientenakten-Software verwendeten – The Health Information Network (THIN), 2) Datenberichte vom UK Health & Social Care Informationszentrum und 3) die Antworten auf Anfragen unter dem Freedom of Information Act (2000).

**Ergebnisse:**

Die dokumentierte Häufigkeit des Demenzsyndroms stieg nach pädagogischen Interventionen nicht an. Pädagogische Interventionen, Leistungszuzahlung und die Aktivitäten der Gedächtniskliniken hatten keine Auswirkung auf die dokumentierte Inzidenz des Demenzsyndroms zwischen 1997 und 2011. Aber es gab Anzeichen für einen Wandel in der Dokumentation von Konsultationen mit demenzkranken Menschen. Es gab keine klaren Auswirkungen der Incentivierung und Gedächtnisklinikaktivitäten in den Prävalenzdaten. Mehr Patienten konsultieren Gedächtniskliniken, aber weniger wurden mit Demenz diagnostiziert.

**Diskussion:**

Es ist nicht klar, warum Inzidenz und Prävalenz des Demenzsyndrom trotz erheblicher Bemühungen nicht angestiegen sind. Dies erfordert weitere Untersuchungen, um Veränderungen von politischen Strategien zu erreichen. Die effektive Leistung der Gedächtniskliniken muss auch weiter untersucht werden.

## Background

Delays in the recognition of dementia syndrome are a common problem across the world, prompting Alzheimer’s Disease International to call in 2011 for earlier diagnosis and intervention [1]. A number of factors appear to influence the delay in recognition of dementia. For example, one recent study from the UK suggested that misattribution of early symptoms to events, experiences, personality or aging delays help-seeking in almost one third of symptomatic people by up to 2 years [2].

A number of attempts have been made in the UK to make recognition of dementia timelier, beginning in 1997 with educational activities aimed at general practitioners (GP) and other primary care professionals, coinciding with the launch of the cholinesterase inhibitors. In 2006 case finding/screening for dementia was added to the GP contract, introducing complex financial incentives calculated through a reimbursement system called the Quality and Outcome Framework (QOF) designed to improve dementia recognition and management. Finally, the implementation of the Dementia Strategy after 2009 saw the development of a network of National Health Service (NHS) memory services with diagnostic and support functions.

A national education programme in 1997 which coincided with the launch in the UK of the cholinesterase inhibitors and memantine had only limited success in recruiting GPs [3] but there was evidence from a randomized controlled trial (RCT) conducted in 2000–2002 that practice-based educational interventions might improve the recognition of dementia and responses to it [4], albeit from a low baseline.

The financial incentives for GPs, introduced in 2006, included payments for keeping a register of patients diagnosed with dementia, for developing care plans and reviewing them in a face to face consultation at least every 12 months and for carrying out appropriate investigations, e.g. full blood count (FBC), calcium, glucose, renal and liver function, thyroid function tests, serum vitamin B12 and folate levels, in those newly receiving a diagnosis of dementia. A financial reward per case diagnosed was included in this package for a short time. Payments also reflected how close GPs were to identifying the expected prevalence of dementia in their practice populations.

The introduction in the UK of financial incentives for earlier diagnosis, and the promotion of memory clinics, remain controversial. These are interventions based on weak evidence. There is no simple, accurate, widely validated primary care screening tool except the mini mental state examination (MMSE) [5], the benefits and harms of screening for dementia are not known, the benefits of memory clinics not established and there is no evidence that earlier diagnosis affects clinician, patient or family decision-making in a beneficial way [6]. Many assumptions have been made about the usefulness of new policies and methods of working, which need to be tested. This article examines the impact of educational initiatives, financial incentives and policy changes on the diagnosis and management of people with dementia in the community, reporting effects on incidence, prevalence and memory clinic activity.

## Material and methods

The primary care research group on dementia at University College London has investigated the impact of educational interventions and policy changes designed to promote timely or earlier diagnosis of dementia syndrome using different methodologies. Here we report data from three sources:

1. Studies that have used aggregated, anonymised data from a network of general practices using the same electronic medical record software, The Health Information Network (THIN). These studies [7–9] have used samples of routinely collected clinical data from THIN; the findings discussed here are from a sample of 1,338,659 patients aged 60 years or over drawn from 476 group practices, collating data from 1995 to 2011.
2. UK Health & Social Care Information Centre (now called NHS Digital) data reports.
3. Responses to Freedom of Information Act requests made by a medical news journal, ‘Pulse’, in 2015.

## Results

### Incidence

**Fig. 1:**
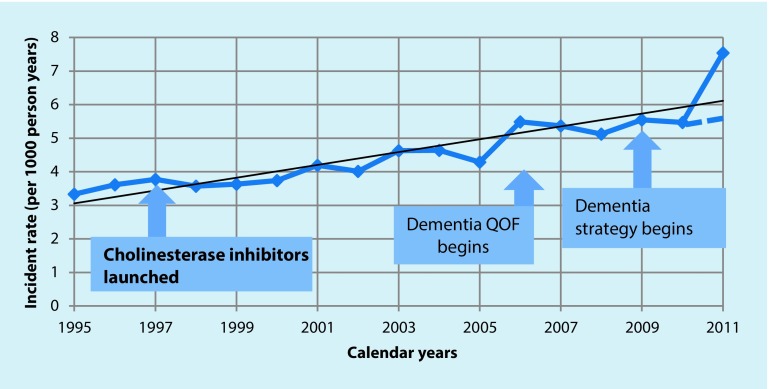
Incident rate of dementia in the THIN database 1995–2011 (*QOF* Quality and Outcome Framework)

Fig. 1 shows the incident rate for dementia in this population, expressed as rate per 1000 person years, using all codes for dementia used in the electronic medical record. There is some variation in incident rates but the trend line over 17 years suggests a slow, steady increase in incident rates with no evident impact following educational campaigns (beginning in 1997) and incentivisation (beginning in 2006). There does appear to be an increase in incident rate after the implementation of the dementia strategy in 2009, which promoted the growth of memory clinics; however, this was an artefact; 2 of the 476 general practices used ‘dementia monitoring’ codes designed to reflect follow-up consultations with people with dementia to document screening individuals for dementia, with the result that 1000 patients received dementia codes without having the diagnosis. Once this had been corrected the incident rate (shown as a dotted line in Fig. 1) reverted to the trend line.

**Fig. 2:**
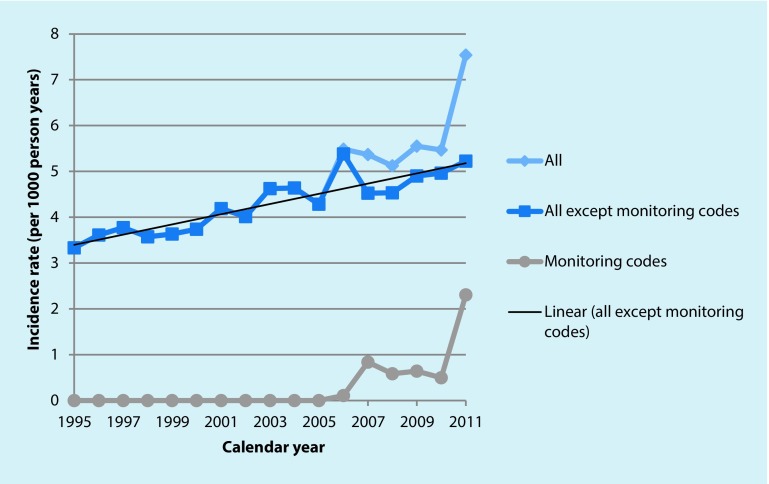
Trends in dementia code use 1995–2011

The financial incentives introduced in 2006 did appear to change the way in which encounters with people with dementia were documented in the electronic medical records (see Fig. 2); a code for ‘dementia monitoring’ was used more often after 2007, with a slight decline in use during 2008–2010 until the sudden but artefactual increase in 2011.

**Fig. 3:**
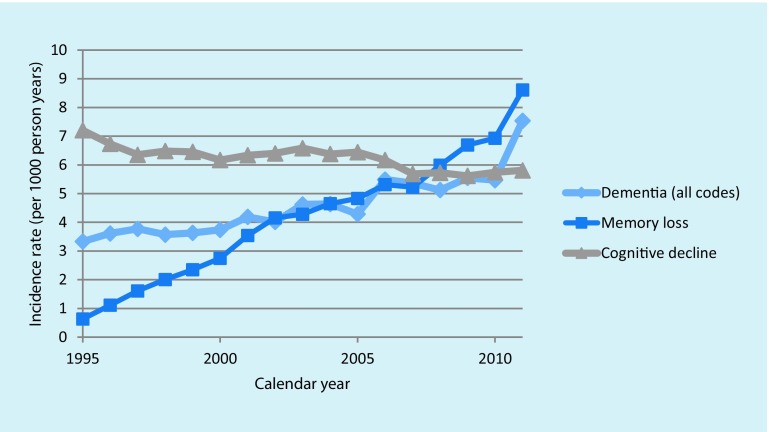
Changes in dementia coding in the years 1995–2011

An increase in the use of a code for ‘memory loss’ is visible in Fig. 3, together with a decline in the use of a ‘cognitive decline’ code.

## Prevalence of dementia

**Fig. 4:**
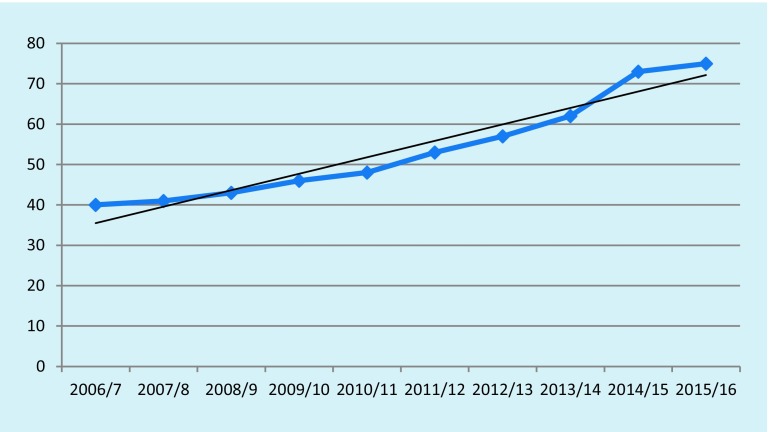
Prevalence of dementia from Quality and Outcomes Framework (QOF) data of selected practices, numbers per thousand population

A similar pattern of slow, steady increase with no interruptions attributable to education, contract change or policy pressures is visible in the prevalence data (reported as numbers with dementia per 1000 population), shown in Fig. 4. This data is derived from the UK Health & Social Care Information Centre [10]. In 2013 the UK Alzheimer’s Society estimated the population prevalence of dementia as 1.3% whilst these data suggest a prevalence of 0.4% in 2006/2007 and 0.73% in 2014/2015.

### Memory clinic activity

**Fig. 5:**
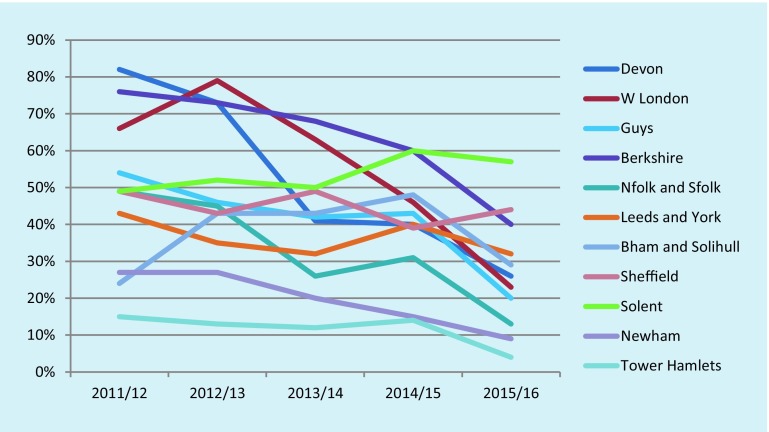
Percentage of patients referred to memory clinics who received a dementia diagnosis (*W London* West London, *Guys* Guys Hospital London, *Nfolk* Norfolk, *Sfolk* Suffolk, *Bham* Birmingham)

**Fig. 6:**
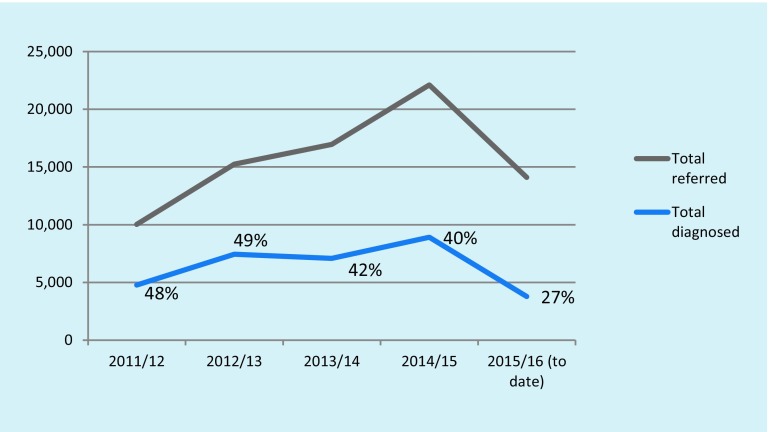
Referrals to 11 memory clinics, and proportions diagnosed as having dementia 2011–2015

There is some evidence that memory clinics are diagnosing fewer cases of dementia. Data obtained from a Pulse magazine Freedom of Information request in February 2016 are shown in Fig. 5. The proportion of those referred to the 11 clinics from which data could be obtained who received a diagnosis of dementia declined between 2011/12 and 2015/16 in all but two clinics. Fig. 6 shows the actual numbers of referrals, and the average proportions receiving a dementia diagnosis.

## Conclusion

Educational interventions did not appear to change the recorded incidence of dementia syndrome, despite evidence from one contemporary trial that they could. This negative finding is supported by the results of a trial of practice-based education in the EVIDEM-ED study [11]. There was no apparent effect of education, incentives or memory clinic activity on the reported incidence of dementia syndrome between 1997 and 2011 but there were signs of change in the documentation of consultations with people with dementia in the electronic medical records, with increases in the use of ‘memory loss’ codes and ‘dementia monitoring’ codes. There was no clear impact of incentivisation and memory clinic activity in prevalence data between 2006/2007 and 2015/2016.

Memory clinics are seeing more patients but fewer are being diagnosed with dementia. This is not surprising given the multiple causes of memory loss and the lack of specificity of memory loss symptoms. The emphasis on memory testing (in general practice) and memory clinics may value subjective memory complaints over other features of dementia syndrome. A Swedish study by Palmer et al. [12] used a 3-stage diagnostic process including a memory question, testing using the MMSE for those with memory complaints and referral of those falling below a predetermined cut-off on the MMSE for a fuller psychometric testing identified 18% of subsequent cases of dementia. More than 50% of people diagnosed with dementia did not have memory problems before diagnosis. As a diagnostic tool, eliciting subjective memory complaints appears to be poor value for ruling in a diagnosis of dementia but good value for ruling out a diagnosis [13].

There are limitations in the use of routinely collected clinical data to judge effects of education, incentivisation and memory clinic performance, in that they cannot reveal changes in clinical practice that might be beneficial to individuals with dementia. Some data sources, such as the memory clinic performance data, are based on small samples and may not be representative. Nevertheless, these data provide insights into the impact that education, incentivisation and promotion of memory clinic may or may not have on the provision of care for people with dementia and prompt the question: after all these efforts, why is there no upturn in incidence or prevalence? It is possible that the educational interventions used did not improve knowledge or clinical confidence in GPs or that the financial incentives to recognise dementia syndrome were too small to change clinical work priorities. Similarly, all of these interventions may act more slowly than we expected and change clinical practice in the medium to long term rather than in the short term. Further investigation is needed to inform future policy changes related to the recognition of and response to dementia.
